# Preliminary Evaluation of a Six-Day Outpatient Psychotherapeutic Training (DIAT) for Anxiety, Depression, and Stress in Adults: A Three-Month Follow-Up

**DOI:** 10.3390/jcm14165806

**Published:** 2025-08-16

**Authors:** Beata Zarzycka, Barbara Krasiczyńska, Marcin Wojtasiński

**Affiliations:** 1 Institute of Psychology, The John Paul II Catholic University of Lublin, 20-950 Lublin, Poland; marcin.wojtasinski@kul.pl; 2 Duc in Altum Psychotherapy Center, 50-329 Wrocław, Poland; barbara.krasiczynska@gmail.com

**Keywords:** psychotherapeutic training, psychotherapy effectiveness, anxiety, depression, stress, well-being, longitudinal study

## Abstract

**Background:** This study presents a preliminary evaluation of a six-day outpatient psychotherapeutic training based on the Duc in Altum Therapy (DIAT) approach, examining its effectiveness in reducing symptoms of anxiety, depression, and stress among adults. Given the growing demand for brief, effective mental health interventions, this format aims to provide intensive support within a condensed timeframe. **Methods**: The intervention consisted of supportive and experiential group therapy delivered over six days and included 109 participants. Outcomes were assessed at three time points—pre-intervention, post-intervention, and three-month follow-up—using the Direct Behavior Rating Scale Items, the Brief Screen for Depression (BSD), and the Perceived Stress Scale (PSS-10). **Results**: Participants reported lower levels of anxiety (B = −1.435, *p* < 0.0001), depression (B = −0.717, *p* < 0.001), and stress (B = −1.882, *p* < 0.001) after training, reflecting statistically significant within-group changes. These improvements were maintained at the three-month follow-up. **Conclusions**: Although the absence of a control group limits causal inference, the findings provide preliminary support for the effectiveness of DIAT as a brief psychotherapeutic intervention. Participants reported lower levels of anxiety, depression, and stress after the training, reflecting statistically significant within-group changes. These improvements were maintained at the three-month follow-up.

## 1. Introduction

Anxiety disorders rank among the most prevalent mental health conditions on a global scale. In 2019, approximately 301 million individuals were affected worldwide, representing roughly 4% of the global population [1]. These disorders frequently originate early in life, have a persistent course, and often coexist with other mental health conditions such as depression and substance use disorders. As a result, they impose significant direct and indirect costs, including heightened utilization of healthcare resources and decreased productivity at work. Individuals afflicted with anxiety disorders commonly encounter difficulties in daily functioning, impairing their capacity to perform social roles and reducing their efficacy in the workplace [2].

Depression and stress are likewise major contributors to the global mental health burden. According to the World Health Organization, depression affects more than 280 million people worldwide and has become the leading cause of disability across age groups [3]. It is associated with significant emotional suffering, functional impairment, and economic loss due to absenteeism and increased healthcare utilization. Clinical evidence indicates that depressive symptoms are highly prevalent among adults, especially during periods of psychosocial stress or chronic illness, and are closely tied to increased sensitivity to negative life events and diminished coping capacity [4,5,6]. Stress, the subjective evaluation of environmental demands as overwhelming or uncontrollable, has been identified as a transdiagnostic risk factor across multiple forms of psychopathology. Elevated levels of perceived stress are strongly associated with the onset, severity, and recurrence of anxiety and depression, making it a clinically relevant indicator of psychological distress (e.g., [7]).

The rising prevalence of anxiety, depression, and stress-related conditions underscores the urgent need for effective and accessible therapeutic interventions. Although conventional long-term individual psychotherapy remains a well-established treatment modality, recent research has validated the effectiveness of group psychotherapy and emphasized the importance of developing short-term group intervention formats [2]. The efficacy of group-based interventions for depression, anxiety, and stress is reinforced by robust clinical trials and meta-analyses. For example, group formats of cognitive-behavioral therapy (CBT) have shown significant effect sizes in alleviating depression and stress, with certain studies demonstrating comparable or superior outcomes relative to individual therapy, particularly in terms of cost-effectiveness and scalability [8]. This is echoed by studies of group interpersonal psychotherapy, which have yielded large, sustained improvements in depression and social functioning across diverse cultural and clinical populations, such as Turkish women with major depression [9] and adolescents [10]. Similarly, group CBT and exposure-based interventions for anxiety disorders and posttraumatic stress disorder (PTSD) have demonstrated meaningful reductions in symptomatology, with a recent meta-analysis highlighting that group CBT for PTSD produces medium to large effect sizes [11,12]. However, within this broader evidence base, experiential and humanistic approaches remain underrepresented, particularly in the treatment of anxiety and stress-related conditions [2,13]. This gap is noteworthy, given that person-centered therapy is inherently relational and thus well-suited to group contexts [14].

Building upon this foundation, the present study aims to evaluate the effectiveness of a six-day outpatient psychotherapeutic training program utilizing the Duc in Altum Therapy (DIAT) methodology for individuals experiencing neurotic, stress-related, and somatoform disorders. The DIAT approach integrates humanistic and experiential techniques to foster emotional expression, self-awareness, and interpersonal connections. By assessing its impact on symptoms of anxiety, depression, and stress, this research seeks to contribute to the development of effective short-term group therapy interventions for individuals experiencing neurotic, stress-related, and somatoform disorders.

### 1.1. Therapeutic Training in Psychotherapy

Therapeutic training constitutes an intensive, short-term group psychotherapy designed to emphasize the development of a profound relationship with oneself and the surrounding environment. Its primary objective is to mitigate psychological distress in the specific areas identified by clients. The training seeks to enhance participants’ inner coherence and self-acceptance, thereby fostering greater internal harmony and more transparent interpersonal relationships. This transformative process is facilitated through self-observation, constructive feedback from the group, individual and group activities, and collaborative exercises. The topics covered during the training are tailored to address the participants’ specific needs and concerns.

Therapeutic training traces its origins to encounter groups developed and facilitated by Carl Rogers in the 1970s [15], which gained significant popularity during the 1980s. Rooted in the humanistic, person-centered tradition, this form of training initially emphasized self-exploration and authentic interpersonal encounters within group settings. In Poland, over subsequent decades, encounter groups have evolved to incorporate a diverse range of psychotherapeutic methods, combining elements from various modalities [16]. This form of short-term group therapy was called therapeutic training. Training involves integrative group processes that combine psychodynamic theories with experiential modalities such as the Gestalt approach and psychodrama. This integration results in a comprehensive group psychotherapeutic process that relies on the interplay of group dynamics, fostering therapeutic alliance, cohesion, and emotional processing among participants [17,18,19]. The transformation from traditional encounter groups to structured interpersonal and therapeutic training has been accompanied by the inclusion of specific interventions delivered by qualified therapists trained to facilitate emotional insight, mentalizing, and corrective relational experiences within the group context. This trajectory highlights the importance of professionally guided processes built upon foundational peer-to-peer therapeutic encounters.

Therapeutic training often serves as a core component of self-exploration in extended professional education programs, including psychotherapy schools, sociotherapy training, and psychological development courses. Meanwhile, there are standalone open therapeutic training opportunities that operate independently of formal programs, providing experiential group formats accessible to a wider audience seeking personal growth or therapeutic support [20]. These training formats often complement individual psychotherapy, serving multiple clinical and educational purposes: as an introductory step to prepare clients for deeper individual therapy, as a parallel intervention to enhance therapeutic gains through group support, or as a concluding process to consolidate therapeutic achievements through shared reflection and relational feedback [21].

The efficacy of group-based therapeutic training modalities can be partly attributed to well-established therapeutic change mechanisms such as the development of a robust therapeutic alliance, group cohesion, and empathic resonance, which promote emotional expression and interpersonal learning. Research indicates that such relational processes constitute significant predictors of positive outcomes in short-term group psychotherapy, emphasizing the importance of the interpersonal and affective quality of group experiences [18,19]. Moreover, the professional facilitator’s role in scaffolding these processes guarantees that training advances from mere group support to focused, corrective emotional experiences and mentalization, thereby enhancing both individual and group therapeutic outcomes [16].

### 1.2. DIAT Method in Humanistic-Experiential Psychotherapy

Founded in 2001 and initially influenced by Gestalt therapy, the Duc in Altum Psychotherapy Center has developed its training program through years of clinical practice. This evolution resulted in the creation of an integrative model named Duc in Altum Therapy (DIAT), after the Center. Over the course of 14 years, DIAT was used in 52 training sessions. In August 2021, a 40 h DIAT course was video-recorded for educational purposes with consent. The first DIAT psychotherapist training started in 2021/22. Ongoing research is assessing DIAT’s effectiveness on mental health and well-being.

The DIAT method is an experiential and integrative therapeutic training model based on a group process. At the core of this approach is the facilitation of interpersonal interactions among participants, the expression of present emotions, and the exploration of significant personal themes within a supportive group environment. The method emphasizes mutual emotional responsiveness, collaborative engagement, conflict management, and processing of transference–countertransference dynamics, creating a rich relational environment that supports therapeutic change [22]. To achieve this, DIAT incorporates psychotherapeutic techniques drawn from multiple modalities, including Gestalt therapy, systemic therapy, psychodrama, emotion-focused therapy (EFT), focusing, symbolic assertiveness, and positive psychotherapy, allowing the approach to meet diverse client needs flexibly [23,24].

Process-experiential therapy has shown outcomes comparable to cognitive–behavioral approaches in treating major depression, with additional benefits for interpersonal functioning and emotion regulation [25]. Furthermore, integrative frameworks that combine experiential, cognitive–behavioral, and systemic elements offer a transdiagnostic basis for treating co-occurring conditions such as anxiety, depression, and stress within a unified therapeutic structure [6,26]. Positioning DIAT within this tradition highlights its theoretical foundations and practical relevance. As a brief, structured group intervention, it is particularly suited to clinical contexts where individuals often present with overlapping psychological and relational difficulties.

Several features set the DIAT method apart from other therapeutic approaches, including integrative ones. DIAT is not based on fixed protocols but on empathy-driven responsiveness to the client’s emotional pain. Techniques from various modalities are used flexibly, depending on the therapist’s attunement to needs, rather than following a set sequence. The experiential phase is personalized and co-created, with the therapist playing a facilitative role. The session rhythm depends on the emotional flow between client and therapist. The group feedback phase is crucial, offering validation, resonance, and a sense of belonging, not just reflection. DIAT focuses on personal transformation, aiming to unlock suppressed emotions, restore meaning, and foster vitality through authentic experiences and relational connection.

DIAT is delivered in small groups of eight to twelve, who undergo an initial screening for suitability, similar to group therapy practices that boost social connection and reduce dropout [27]. The 40 h training is divided into several four-hour sessions, with breaks, to support emotional processing, based on research on emotional arousal in experiential interventions [28]. Two licensed psychotherapists co-facilitate. Off-site locations are preferred to create a contained community, enhancing cohesion and safety [27].

The group development process within DIAT aligns with well-established stages essential for therapeutic efficacy: (1) an initial phase of group formation involving contractual agreements and establishment of safety and trust; (2) a middle phase of active emotional and experiential work through assumed group roles and collaboration; and (3) a final phase focused on secure disengagement and reintegration, enabling participants to consolidate gains and transfer skills to everyday settings [29]. This structured progression enhances psychological safety and supports sustainable emotional and social adaptation.

Within the individual therapeutic process, DIAT describes three phases. The first, “leaf raking,” involves a client interview using tools like the “pain compass” to identify distress and set themes for experiential work. This step aids emotional processing and mentalization [24]. The second phase involves the deep processing of blocked or painful material, tailored to the client’s readiness, utilizing modalities such as psychodrama, systemic interventions, and emotion-focused techniques. The therapist’s skills are vital for optimizing affect regulation and fostering corrective experiences [24,27]. The final phase involves group and therapist feedback to integrate insights and reduce anxiety, deepening emotional processing and supporting lasting change through reflection [23]. An example of a therapeutic process conducted within the DIAT framework is provided in Supplementary Material S6.

### 1.3. Present Study

This study aimed to evaluate the effectiveness of a six-day psychotherapeutic training program (DIAT) in reducing symptoms of anxiety, depression, and stress among adult clients in Poland. A longitudinal design was used, with three assessment points: before the intervention (baseline), immediately after it ended, and three months later. Responding to the increasing demand for accessible and scalable mental health interventions, the study examined whether an intensive, yet short-term, therapeutic format could produce lasting improvements in psychological well-being. Using this framework, we developed the following hypotheses about the changes in symptoms over time.

**Hypothesis** **1:** *Participants will report a reduction in symptoms of anxiety, depression, and stress across the three assessment points, reflecting a general within-group improvement over time.*

**Hypothesis** **2:** *The symptom reduction observed immediately after the intervention will be sustained at the three-month follow-up, indicating the potential durability of changes within this sample.*

**Hypothesis** **3:** *The pattern of symptom change will be relatively consistent across participants, regardless of their baseline severity, suggesting a broadly similar trajectory of change across the group.*

## 2. Method

### 2.1. Participants and Procedure

A total of 109 individuals participated in the study, comprising 91 females and 16 males, with 2 participants not disclosing their gender. The age range of participants varied from 21 to 62 years, with a mean age of 37.55 years (*SD* = 7.53). Most participants lived in urban areas (*n* = 93), while fewer resided in rural areas (*n* = 16). The training took place in a private setting, and all participants independently funded their involvement. Despite the small sample size, the results remain comparable to those of other exploratory studies evaluating similar therapies or conditions (e.g., [17,30]).

The study sample consisted of adults who had been accepted into a six-day therapeutic training program through the standard qualification procedure of the Duc in Altum Psychotherapy Center. Recruitment into the training groups followed routine clinical practice and was conducted independently of the study. As part of the qualification process, all applicants completed a written intake form and participated in an individual consultation with a therapist, who assessed their psychological readiness and suitability for the intensive group format. The inclusion criteria included being at least 18 years old, demonstrating psychological stability, and possessing sufficient interpersonal skills to participate in emotionally focused group sessions. Individuals exhibiting active psychotic symptoms (e.g., hallucinations, delusions, disturbances in contact), severe social withdrawal, or other contraindications for group therapy were excluded. No systematic records were kept regarding the total number of applicants or the number who did not meet the inclusion criteria, as group formation followed routine scheduling and was not influenced by the research procedures.

Individuals who were accepted into the training (N = 109) were subsequently invited to participate in the study, and all provided informed consent. They were informed about the study’s purpose and procedures, which included that participation was voluntary and anonymous. Before training, participants completed demographics and three questionnaires on anxiety, depression, and stress (T1). They then attended a six-day psychological training (DIAT). After the training (six days later), they completed the same questionnaires again (T2). The third set was collected online three months later (T3). No other interventions occurred during this period. All attended every DIAT session, with no dropouts or adverse events. The Ethics Committee at the first author’s university approved the study protocol on 4 April 2025 (ref. KEBN 27/2025).

A high retention rate of 98% was achieved, likely supported by the structured, time-limited format of the six-day intervention. Additionally, participants’ understanding of this study’s relevance and purpose may have contributed to sustained engagement across all assessment points.

### 2.2. Treatment Conditions

In the intervention setting, participants participated in a six-day therapeutic training program, attending twice-daily group therapy sessions, each lasting four hours. A break was provided during each session. The intervention was founded on an integrative approach, incorporating humanistic-experiential psychotherapy. Although the structure of the intervention was manualized, it was not prescriptive in terms of content. The principal therapeutic objectives comprised facilitating emotional expression, processing personal meanings, fostering deeper respect and care for oneself and others, and promoting mutual support. The intervention did not emphasize the management of anxiety, depression, or stress. Throughout each session, participants retained the autonomy to select the topics they wished to discuss, commonly including themes such as experiences of loss and bereavement, challenges in close relationships, spirituality and life goals, social networks, and intimate relationships.

All sessions were organized by the Duc in Altum Psychotherapy Center and were characterized as interactive experiences where participants lived collectively and shared meals. Each group consisted of 10 to 12 individuals, all of whom attended every session and remained fully engaged throughout the entire training period. Each group session followed a structured format: (1) an opening round with questions such as, “How do you start your day? How do you feel about yesterday’s experiences? How did the night go? Is there anything you want to share with the group or a specific individual?”; (2) a volunteer selects an individual to introduce a personal topic; (3) interview, experiential activity, and feedback, where other participants share their insights; (4) a concluding round to review the day. In the interview, clients are encouraged to explore their emotions and understand their meanings, with a particular focus on bodily sensations and their associated feelings. After identifying the pain and the areas needing change, clients receive experiential insights and are invited to express repressed emotions symbolically. This process sometimes reveals insights into their family dynamics and allows for alterations in their lived experiences. The intervention addressed internal conflicts and the inner critic while guiding clients in self-soothing and self-protection techniques. By examining the organization of the participants’ internal self-configurations, the training fostered a caring attitude toward themselves, encouraged self-dialog, and heightened awareness of maladaptive behavioral patterns. The EFT interventions were incorporated, particularly those aimed at addressing emotional pain and enhancing agency and personal dignity. Frequently, through this experiential approach, clients articulate themselves in new ways for the first time, facilitating the formation of new neural connections [31]. Therapists provided guidance on recognizing and expressing one’s authentic emotions. Feedback sessions helped consolidate these emotional shifts, often generating resonances of shared experience that encouraged further group reflection. Such engagement can also inspire further discussions with other participants. Notably, across sessions, collective emotional release was observed when one participant accessed deep emotions symbolically; others frequently experienced parallel therapeutic transformations. This phenomenon aligns with well-established mechanisms of change in group therapy—such as emotional mirroring, group cohesion, and corrective interpersonal experiences—known to enhance therapeutic outcomes [32].

Participants had the freedom to choose how to spend each evening, whether resting alone, engaging in informal conversations, or praying if they wished. The chapel was kept open for personal meditation or prayer. One evening was dedicated to collective meditation centered on the Talitha cum icon. Spiritual elements were included in the DIAT training as optional support, mainly to address participants’ individual needs when spirituality was meaningful to them. These elements were not essential but aimed to enhance personal reflection and emotional integration for those who found spiritual symbols or practices beneficial.

Treatment consistency was maintained by ensuring all therapists followed a shared therapeutic framework based on the DIAT method, which includes three main components: an initial interview, an experiential group process, and a feedback and integration phase. While the method allows for flexibility and responsiveness to individual client needs, these common elements provide a clear structure across groups. To ensure fidelity, each day ended with a supervised session led by a DIAT-trained supervisor who was not directly involved in facilitating that specific group. During supervision, compliance with DIAT principles was checked, and the progress of the process was monitored.

### 2.3. Therapists

The psychotherapeutic groups were led by experienced clinicians trained in a humanistic-experiential approach, including person-centered, experience-centered, and emotion-focused modalities, such as Gestalt therapy. Each therapist had completed a four-year psychotherapeutic training, undergone specialized instruction in DIAT methodology, and held certification in experiential psychotherapy. All facilitators had over 15 years of clinical experience, and the second author provided direct supervision and ongoing DIAT training. Every session was conducted with care and professionalism, ensuring adherence to best practice standards. To enhance transparency, we acknowledge that the second author, as a developer of the DIAT method, served as a co-facilitator in each group. To reduce potential allegiance bias, all groups were co-led by experienced therapists trained in the method, and daily supervision was conducted by an independent DIAT-trained supervisor who was not involved in delivering the intervention.

### 2.4. Methods

To minimize participant burden during the intensive therapeutic process while maintaining sensitivity to clinically meaningful change, brief screening instruments were selected.

#### 2.4.1. Anxiety

The three-item Direct Behavior Rating Scale Items Scale (DBR-SIS) [33] was used to assess the anxiety levels of the participants. The DBR-SIS comprises three items on a Likert scale, which reflect the social (e.g., I am concerned about others’ perceptions), cognitive (I experience restlessness), and physiological (I feel nervous) dimensions of anxiety. Responses to these items were provided on a 10-point Likert scale, with endpoints categorized as 1 (no anxiety) and 10 (very high anxiety). Elevated scores correspond to increased levels of anxiety. In this study, Cronbach’s α was 0.86.

#### 2.4.2. Depression

The Brief Screen for Depression (BSD), a 4-item tool, was used to assess the level of depressive symptoms in respondents [34]. For instance, respondents were asked how often they felt hopeless, helpless, pessimistic, intensely worried, or unhappy in the last two days. They rated this first item on a scale from 1 (not at all) to 5 (all of the time), while items 2 through 4 were rated on a scale from 1 to 10. The BSD score is calculated by summing the scores of items 2 to 4 and multiplying the score from item 1 by 4. Thus, the overall score reflects four times the value of item 1 plus the sum of the other items. A higher total indicates increased levels of depression. In this study, Cronbach’s α was 0.71.

#### 2.4.3. Stress

The 10-item Perceived Stress Scale (PSS-10) [35], adapted into Polish [36], was used to assess the frequency of perceived stress experienced over the past month (e.g., In the last month, how often have you felt nervous or stressed?). Participants answered the items using a 4-point Likert scale, with response anchors ranging from 0 (never) to 4 (very often). Higher scores indicate increased levels of anxiety. In this study, the Cronbach’s alpha coefficient was calculated to be = 0.90.

### 2.5. Statistical Analysis

**Figure 1:**
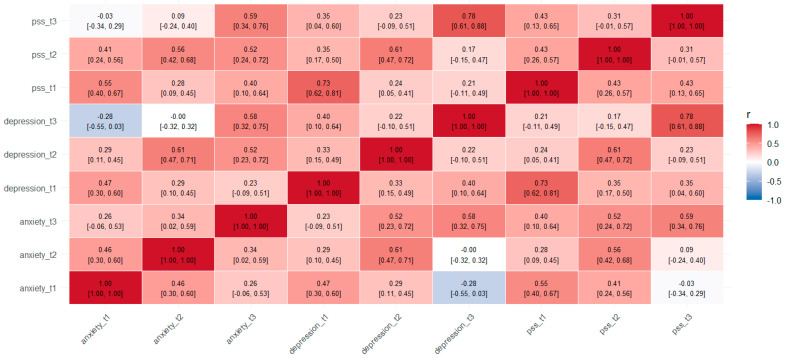
Pearson correlation heatmap of anxiety, depression, and perceived stress (PSS) across three time points (T1, T2, T3). Darker colors indicate stronger correlations. The matrix displays both within- and between-construct relationships across time, illustrating moderate to strong associations among symptoms.

We conducted all analyses in R 4.2 [37] using the lavaan package (v. 0.6–12) [38] with full-information maximum likelihood (FIML) under a missing-at-random assumption [39,40]. Specifically, we performed the following steps: descriptive statistics, 95% confidence intervals, and effect sizes. For each outcome (anxiety, depression, and stress) at T1, T2, and T3, we computed means, standard deviations, sample sizes, and 95% confidence intervals (CIs) [41]. Within-subject Cohen’s *d* was calculated for T1→T2 and T1→T3 changes [42]. Results are reported in Table 1; missing-data summaries appear in Supplementary File S1. Pearson correlations (and their 95% Fisher *z* confidence intervals) [43,44] were computed among all nine observed scores, which are presented in Figure 1.

For each outcome, we fit a two-factor LGM (intercept loadings fixed at 1; slope loadings fixed at 0, 1, 2). Key fit indices (CFI, TLI, RMSEA [90% CI], SRMR) are reported in Supplementary File S2, and full parameter estimates in S3 [45]. Variances and covariances of the latent factors are provided in Supplementary File S4. Given that LGMs via FIML assume multivariate normality (or at least approximate it), we added a routine check of skewness, kurtosis, and Shapiro–Wilk tests for each observed variable at T1, T2, and T3 (see Supplementary File S5). Because several variables showed modest departures from normality, we re-ran all growth models using the robust maximum likelihood estimator (MLR) in lavaan, which produces standard errors and chi-square tests that are robust to non-normality. The substantive patterns of parameter estimates, fit indices, and confidence bands were similar under MLR, confirming that our conclusions are not driven by minor violations of normality.

## 3. Results

### 3.1. Descriptives and Correlations

Table 1 displays, for each outcome at T1, T2, and T3, the sample size (N), mean (SD), 95% confidence interval, and within-subject Cohen’s d for the adjacent change (T1→T2) or follow-up change (T1→T3). For example, anxiety fell from M = 5.22 (SD = 2.26), 95% CI [4.79, 5.65] at T1 to M = 2.86 (SD = 1.92), 95% CI [2.49, 3.23] at T2 (d = 1.07), and remained lower at T3 (M = 3.67, SD = 1.86, 95% CI [3.07, 4.26]; d = 0.69 from T1). Depression and stress showed analogous large pre–post and moderate pre-follow-up effect sizes (see Table 1).

To describe how the three constructs varied together within and across waves, we then calculated a Pearson correlation matrix (Figure 1). The heatmap shows pairwise correlations for each time-specific variable, with darker shades indicating stronger connections. Significant relationships were found both within and between symptom domains over time. Moderate links were seen for anxiety across measurement points (T1–T2 *r* = 0.46; T2–T3 *r* = 0.34), as well as for depression (*r* = 0.40–0.61) and perceived stress (*r* = 0.43; 0.31). Cross-sectional correlations were also significant, such as anxiety with depression (T1 *r* = 0.47) and anxiety with stress (T1 *r* = 0.55). Importantly, lagged correlations showed that anxiety at T1 predicted stress at T2 (*r* = 0.41) and depression at T2 (*r* = 0.29). These results highlight the interconnectedness of these symptom domains and support the use of latent growth models to examine their changes over time.

### 3.2. Latent Growth Models

Separate two-factor latent growth models were fitted for each outcome (anxiety, depression, PSS) in lavaan (R 4.2) using full-information maximum likelihood (FIML). For each model, the intercept factor loadings were fixed at 1 across T1–T3 (i = 1 · T1 + 1 · T2 + 1 · T3) and the slope factor loadings at 0, 1, and 2 (s = 0 · T1 + 1 · T2 + 2 · T3). Model fit was evaluated with CFI, TLI, RMSEA (with 90% CI), and SRMR (see Supplementary S2). Full output—including factor loadings, latent variances, covariances, and parameter estimates—is provided in Supplementary S3, and a summary of latent factor variances and covariances in Supplementary S4.

#### 3.2.1. Trajectory of Anxiety

The results show a significant decrease in anxiety across the three measurement points, with a steady decline following the psychotherapy treatment. Specifically, the initial anxiety levels, indicated by the intercept (i), are significantly above zero (Estimate = 4.922, SE = 0.27, *p* < 0.0001). This indicates that participants started the study with a noticeable level of anxiety, emphasizing the importance and need for the therapeutic intervention for this group.

**Figure 2:**
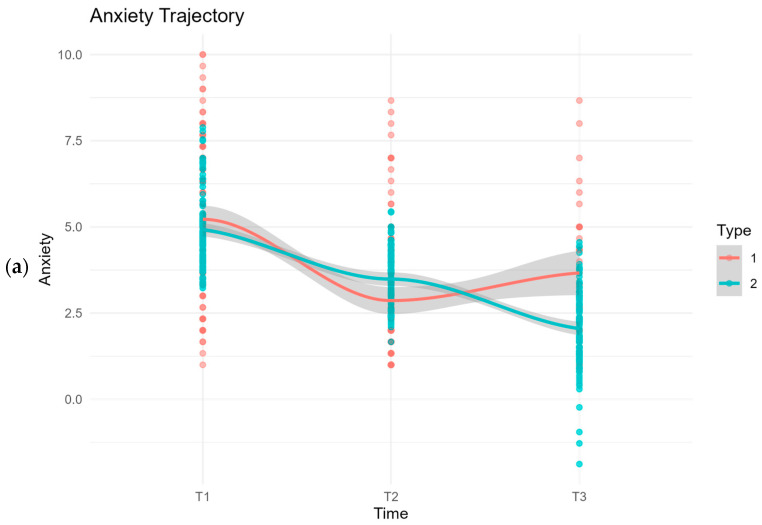

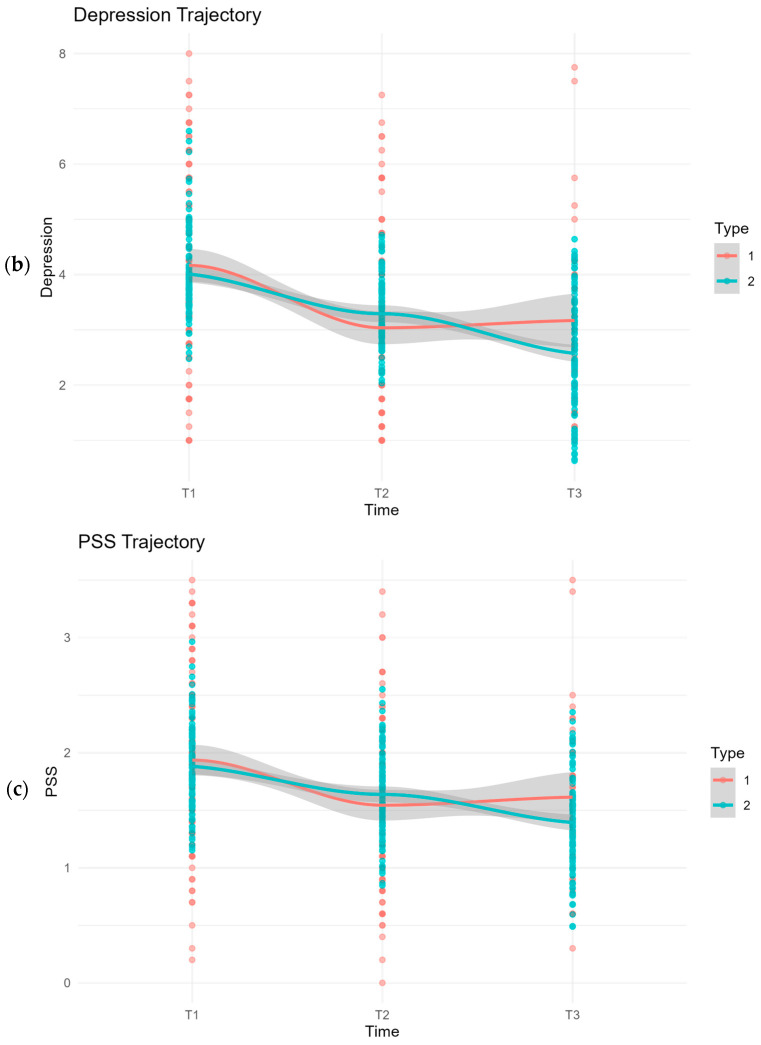
Latent growth model (LGM) illustrating the trajectory of (**a**) anxiety, (**b**) depression, and (**c**) stress (PSS) levels across three measurement points: pre-intervention (T1), post-intervention (T2), and three-month follow-up (T3). Red circles and line represent the observed mean scores (Type 1); blue circles and line represent the model-predicted trajectories (Type 2), with shaded bands indicating 95% pointwise confidence intervals around the predicted means.

The negative slope (Estimate = −1.435, SE = 0.237, *p* < 0.0001) indicates a significant decrease in anxiety levels over time. The consistent reduction observed across the three time points (pre-training, post-training, and three months later) suggests that the benefits of the therapy were not only immediate but also maintained during the follow-up period. Figure 2a depicts the trajectory of anxiety levels measured at three different time points.

The analysis of variances in initial anxiety levels and the change rate reveals some interesting insights. The variance for the intercept (i) is 1.841 (*p* = 0.268), and for the slope (s), it is −0.961 (*p* = 0.396). These non-significant variances indicate limited differences among individuals regarding their starting anxiety levels and the rate at which their anxiety decreased over time. In other words, the participants in this study had similar initial anxiety levels and experienced comparable rates of anxiety reduction through the intervention. This homogeneity suggests that psychotherapy had a broadly uniform effect across the sample rather than varying greatly from person to person.

Furthermore, the covariance between the intercept (i) and slope (s) is −0.031, with a *p*-value of 0.982, indicating that this relationship is not statistically significant. This non-significant covariance suggests that initial anxiety levels do not strongly predict the trajectory of change over time. In other words, whether a participant began with higher or lower anxiety did not significantly influence the pattern of symptom change observed across the three assessment points.

Overall, the results indicate a consistent pattern of change in anxiety scores across measurement points, with observed decreases maintained at follow-up. The limited variability between individuals and the non-significant covariance between baseline anxiety and change trajectory suggest a relatively uniform response pattern within the sample. These preliminary findings point to the potential relevance of the intervention across different initial anxiety levels.

#### 3.2.2. Trajectory of Depression

The results show a substantial decrease in depression across the three measurement points, with a steady decline following the psychotherapy intervention. The initial depression levels, indicated by the intercept (i), are significantly above zero (Estimate = 4.009, SE = 0.148, *p* < 0.001). This indicates that participants started the study with notable depressive symptoms, emphasizing the importance of the therapeutic intervention in addressing these symptoms.

The negative slope (Estimate = −0.717, SE = 0.119, *p* < 0.001) indicates a significant decrease in depression levels over time. The consistent decline in depression levels across the three time points (pre-training, post-training, and three months later) shows that the benefits of the therapy were not only immediate but also lasted during the follow-up period. Figure 2b shows the path of depression levels across the three measurement points, highlighting the significant changes and trends observed over time.

The analysis of variances in initial depression levels and their rate of change reveals some interesting insights. The variance for the intercept (i) is 0.083 (*p* = 0.888), and for the slope (s), it is −0.688 (*p* = 0.110). These non-significant variances indicate limited differences among individuals in their starting depression levels and how quickly their depression decreased over time. In other words, the participants in this study started with similar depression levels and showed similar rates of symptom reduction through the intervention. This consistency suggests that the psychotherapy had a generally uniform effect across the sample, rather than varying significantly between individuals.

Furthermore, the covariance between the intercept (i) and slope (s) is 0.692 with a *p*-value of 0.139, indicating that this relationship is not statistically significant. This non-significant covariance suggests that initial depression levels do not strongly predict the trajectory of change over time. In other words, whether a participant began with higher or lower depression did not significantly influence the pattern of symptom change observed across the three assessment points.

Overall, these results demonstrate a consistent decrease in depression scores over the measurement points. The small differences among individuals and the non-significant relationship between initial depression and the rate of change indicate a generally uniform response within the sample. These early findings suggest that the intervention could be relevant for participants with varying initial depression levels.

#### 3.2.3. Trajectory of Stress

The results show a significant decrease in perceived stress across the three measurement points, with a steady decline following the psychotherapy intervention. The initial stress levels, represented by the intercept (i), are significantly higher than zero (Estimate = 1.882, SE = 0.069, *p* < 0.001). This indicates that participants started the study with considerable stress, emphasizing the need for the therapeutic intervention to address these symptoms.

The negative slope (Estimate = −0.244, SE = 0.051, *p* < 0.001) shows a significant decrease in perceived stress levels over time. The steady decline in stress levels across the three time points (pre-training, post-training, and three months later) shows that the therapy’s benefits were not only immediate but also lasted through the follow-up period. Figure 2c displays the trajectory of anxiety levels measured at these three distinct time points.

Analyzing variances in initial stress levels and their rate of change reveals some interesting insights. The variance for the intercept (i) is 0.146 (*p* = 0.196), and for the slope (s) it is −0.076 (*p* = 0.324). These non-significant variances suggest limited variability among individuals regarding their starting stress levels and the rate at which their stress decreased over time. In other words, the participants in this study had relatively similar initial stress scores and experienced similar rates of stress reduction through the intervention. This homogeneity indicates that the psychotherapy intervention had a generally uniform effect across the sample, rather than varying greatly from person to person.

Furthermore, the covariance between the intercept (i) and slope (s) is 0.063, with a *p*-value of 0.465, indicating that this relationship is not statistically significant. This non-significant covariance suggests that initial stress levels do not strongly predict the rate of stress reduction over time. Practically, whether a participant started with a higher or lower stress level did not significantly influence how quickly or slowly their stress symptoms decreased.

Overall, these results indicate a steady decline in stress levels across all measurement points. The minimal variability among individuals and the non-significant covariance between initial stress levels and the rate of change suggest that the intervention was consistently helpful, making it a promising option for those experiencing stress.

## 4. Discussion

The results of this study provide preliminary support for the potential benefits of the six-day experiential psychotherapy training (DIAT) in reducing symptoms of anxiety, depression, and stress. Statistically significant improvements were observed across all three assessment points, with symptom reductions evident immediately after the intervention and sustained at the three-month follow-up, thereby supporting Hypotheses 1 and 2. Moreover, the decrease in symptoms was relatively homogeneous across participants, regardless of their initial symptom severity, which lends support to Hypothesis 3. The observed effect sizes exceeded the conventional threshold for clinical significance (*d* ≥ 0.80) [46], suggesting that the improvements were not only statistically meaningful but also likely to be perceptible and beneficial in everyday functioning.

These findings reinforce a growing body of research supporting the utility of short-term group psychotherapies, especially in the reduction in anxiety and depressive symptoms. Meta-analytic and clinical studies illustrate that short-term group interventions, often grounded in dynamic or cognitive-behavioral principles, yield significant symptom reductions in disorders such as depression and anxiety [2,17]. For example, Bros et al. [17] demonstrated that short-term dynamic group psychotherapy produced significant improvements in depression and anxiety symptom ratings when compared to standard care in a primary care population. Similarly, Mielimąka et al. [30] observed substantial decreases in trait and state anxiety in adults treated with intensive, short-term group psychotherapy for neurotic and personality disorders. Our study aligns with these results, extending their implications by integrating supportive and experiential elements within a six-day outpatient format.

The rationale for the potential benefits of group-based experiential training can be understood in light of established mechanisms of therapeutic change. Group therapy offers a unique setting where interpersonal processes, such as empathic resonance, social learning, and corrective emotional experiences, drive significant personal transformation. The DIAT intervention utilized these group dynamics by encouraging mutual sharing, emotional feedback, and real-time interpersonal interactions. Extensive research shows that strong group cohesion, a solid patient-rated alliance, and feedback-rich environments are linked to better outcomes in group therapy [19,47]. Additionally, these group phenomena allow individuals to observe and participate in others’ healing processes, which further promotes insight and emotional development [19].

A further domain of change in the DIAT intervention pertains to the inclusion of professional psychotherapeutic techniques designed to access and resolve unfinished emotional experiences, promote the mentalization of personal schemas, and facilitate the expression of underlying needs. These approaches align with process-experiential and schema-centered models of psychotherapy, which are particularly effective in fostering self-understanding, emotion regulation, and adaptive interpersonal functioning [25,47]. Watson et al. [25] found that process-experiential therapies led to significant improvement in self-reported interpersonal problems and emotional coping, even when compared directly against cognitive-behavioral therapy for depression. Tschacher et al. [47] further elucidated that clarification and emotional activation within group processes serve as key mechanisms for enduring clinical change.

From a practical standpoint, the DIAT training demonstrates several salient advantages. Its brief duration, cost-effectiveness, and group-based delivery make it an attractive alternative or adjunct to long-term individual therapy. The literature consistently notes that short-term group interventions can often yield rapid improvements, conserve healthcare resources, and be more accessible for patients who may not engage in or require extended psychotherapeutic support [17,48,49]. Lorentzen et al. [49] and Abbass [48] showed that short-term group therapies are generally as effective as longer-term alternatives for most outpatients, except perhaps for those with severe personality pathology or chronic distress who may benefit more from extended treatment. Given the homogeneous benefit observed in the current sample, regardless of initial symptom severity, the DIAT approach appears to be widely applicable—a finding that aligns with evidence suggesting that short-term group therapies can be efficacious across diverse clinical profiles.

Notably, while brief intensive group programs such as DIAT generate immediate and sustained symptomatic relief, they are not positioned to replace comprehensive long-term interventions. Rather, they function as efficient entry points, catalysts for deeper psychotherapeutic work, or transitional supports to optimize social functioning and self-reflection [17,48,49]. The potential for group-driven modalities to expedite corrective change and foster mutual support underscores their relevance within stepped-care models and contemporary mental health systems [50].

This study is the first empirical evaluation of the DIAT method, enhancing understanding of brief experiential group therapies. A key limitation is the lack of a randomized control or comparison group, making it hard to attribute changes solely to DIAT. This allows for alternative explanations, such as regression to the mean, spontaneous remission, expectancy, or placebo effects, as well as the passage of time, all of which can reduce symptoms [4,6]. Research in psychotherapy shows that factors such as therapeutic alliance, social support, or participation in structured groups can produce improvements even without active treatment [5]. Additionally, symptom changes may result from natural life events or recovery, not treatment, without a comparison group [51]. Future randomized controlled trials are essential for distinguishing treatment effects from confounding factors and establishing evidence for brief, integrative group interventions.

### 4.1. Limitations

Several limitations should be addressed. First, although the sample size of 109 participants is comparable to that of similar psychotherapeutic intervention studies [17,30], this relatively modest number may limit the statistical power and robustness of subgroup analyses. Additionally, the predominance of female participants restricts the generalizability of findings; women generally demonstrate a greater proclivity toward seeking psychological help and engaging in therapy, which may have influenced the treatment response [50]. Further research should recruit more gender-balanced samples to validate the applicability of DIAT across sexes.

Second, the absence of a control or comparison group precludes definitive causal attributions. The inclusion of randomized control groups, such as waitlist controls, active/placebo interventions, or standard individual therapies, would provide stronger evidence regarding DIAT’s specific effects [17,49].

Third, reliance on self-report instruments—namely the Direct Behavior Rating Scale Items, BSD, and PSS-10—to assess outcomes introduces potential biases related to subjective perception, social desirability, and response styles. Incorporating objective physiological measures (e.g., heart rate variability, salivary cortisol), observer ratings, or qualitative interviews could provide a more comprehensive and nuanced evaluation of symptom changes and experiential aspects of therapy [47].

Fourth, the lack of random allocation to treatment introduces selection bias. Participants self-selected and self-financed enrollment in DIAT, likely reflecting higher motivation or self-efficacy, which may have positively contributed to treatment outcomes; however, this limitation may also reduce ecological validity for less motivated populations [48]. This financial self-selection may also reflect a treatment-seeking or psychologically minded subgroup, potentially reducing the generalizability of the results to broader or more diverse populations.

Fifth, due to the group-based format of the intervention, there is a possibility that social conformity or group contagion effects influenced participants’ self-reported improvements. Shared emotional dynamics, perceived group norms, or the desire to align with others’ progress could have affected outcome ratings. Future studies may consider incorporating observer ratings or behavioral indicators to account for such interpersonal influences more accurately.

Sixth, the scope of outcome variables focused primarily on symptom reduction (anxiety, depression, stress). It did not encompass broader domains of mental health, such as positive affect, overall well-being, or quality of life, all of which are critical markers of recovery and resilience [6]. Future studies should integrate multidimensional outcome assessments to gain a more comprehensive understanding of DIAT’s impact.

Seventh, the three-month follow-up period, while valuable, is relatively brief. Longer-term follow-ups are crucial for ascertaining the durability and stability of therapeutic gains, as well as for detecting potential relapse or symptom recurrence [52]. Testing the generalizability of the DIAT method across diverse demographic, cultural, and clinical populations will further establish its universality and ecological validity.

Finally, two additional limitations are worth noting. Each latent growth model was estimated using only three measurement waves, resulting in just-identified models and providing global fit indices (CFI, TLI, RMSEA, SRMR) that cannot be reliably used to assess model misspecification. Although we report intercept, slope, variance, and covariance estimates, readers should interpret these parameters without the usual confirmatory fit validation—future studies should include more measurement points or multiple indicators per wave to allow for formally testable growth models. While missingness was minimal at T2 (<2%), attrition by the three-month follow-up was significant (up to ~60% for some outcomes). Although FIML under a Missing-At-Random assumption uses all available data, the smaller effective sample size at T3 limits statistical power and could bias estimates if dropout was non-random. Replication with larger samples or more robust retention strategies is recommended to verify the durability of treatment effects.

### 4.2. Clinical Implications

This study’s findings underscore that short-term, intensive psychotherapy training, such as the six-day DIAT format, can offer an effective, resource-efficient treatment option for adults experiencing anxiety, depression, and stress-related symptoms. Its condensed timeframe makes it particularly suited for populations requiring accelerated therapeutic engagement, including individuals in crisis or those with limited access to long-term care [17,30].

The group experiential format capitalizes on well-established change mechanisms, such as therapeutic alliance, cohesion, and empathic resonance, within group processes, which have been previously shown to positively mediate treatment outcomes in group psychotherapy contexts [9]. These elements, combined with integrative interventions drawn from Gestalt therapy, experiential emotion-focused therapy (EFT), psychodrama, and systemic therapy, provide a rich therapeutic milieu that fosters emotional processing, mentalization, and interpersonal learning [14].

Furthermore, short-term group therapies, which enable the simultaneous treatment of multiple individuals, are cost-effective and scalable approaches within healthcare systems, particularly in primary care or community mental health settings [17,48]. They can function as either stand-alone interventions or as adjuncts and preparatory steps for longer-term individual psychotherapy, accelerating therapeutic progress and social functioning [53].

Importantly, the observed symptom improvements were not only immediate but also lasted over three months, supporting the clinical usefulness of this brief intervention beyond short-term relief. This durability increases the practical value of DIAT as a viable treatment option in stepped-care or time-limited service models.

### 4.3. Future Research Directions

Future research should seek to replicate these results, employing randomized controlled methodologies to more rigorously delineate treatment effects. Broadening the sample to include a more diverse demographic and clinical spectrum would increase the generalizability of the results. Furthermore, research could examine specific elements of the DIAT method—such as experiential techniques or feedback structures—that are most closely linked to clinical improvement. Longitudinal studies with extended follow-up periods would be instrumental in assessing the durability and potential delayed benefits of such interventions. Lastly, qualitative research could shed light on subjective experiences and participants’ mechanisms of change, providing a deeper understanding of how the DIAT method facilitates therapeutic progress.

## 5. Conclusions

This study on the six-day outpatient psychotherapeutic training using the DIAT model provided preliminary support for the potential benefits of the DIAT intervention in reducing anxiety, depression, and stress symptoms, with the effects persisting at a three-month follow-up post-intervention. Despite methodological limitations related to sample composition, lack of randomization, and control conditions, this study contributes meaningful preliminary evidence supporting the value of brief experiential group interventions. The findings encourage further rigorous investigations, including randomized controlled trials, longer follow-ups, and diversified samples, to consolidate the evidence base for DIAT and elucidate its mechanisms of action. Broadening outcome measures to capture positive mental health indices and incorporating objective assessments will deepen understanding of its therapeutic impact. Given its efficiency, theoretical integrativeness, and promising clinical utility, the DIAT psychotherapeutic training represents a viable and innovative modality poised to complement current therapeutic offerings across various clinical and community settings.

## Figures and Tables

**Table 1:** Descriptive statistics for anxiety, depression, and stress at T1, T2, and T3.

Variable	Time	N	Mean (SD)	95% CI	dT1→T2	dT1→T3
**Anxiety**	T1	108	5.22 (2.26)	[4.79, 5.65]	—	—
	T2	107	2.86 (1.92)	[2.49, 3.23]	1.07	—
	T3	40	3.67 (1.86)	[3.07, 4.26]	—	0.69
**Depression**	T1	108	4.17 (1.58)	[3.87, 4.47]	—	—
	T2	107	3.04 (1.54)	[2.74, 3.33]	0.63	—
	T3	40	3.17 (1.58)	[2.64, 3.70]	—	0.84
**Stress**	T1	108	1.94 (0.73)	[1.79, 2.09]	—	—
	T2	107	1.54 (0.68)	[1.40, 1.68]	0.52	—
	T3	93	1.61 (0.69)	[1.46, 1.75]	—	0.61

## Data Availability

Data available in a publicly accessible repository. The script and dataset are available in the repository at https://osf.io/bdawf/?view_only=66017d108ec5468e8b7f787e5a1ab942.

## Supplementary Materials

The script and dataset are available in the repository at https://osf.io/bdawf/?view_only=66017d108ec5468e8b7f787e5a1ab942, (accessed on 16 July 2025).
